# Peer instruction improves comprehension and transfer of physiological concepts: a randomized comparison with self-explanation

**DOI:** 10.1007/s10459-018-9858-6

**Published:** 2018-10-20

**Authors:** Marjolein Versteeg, Floris M. van Blankenstein, Hein Putter, Paul Steendijk

**Affiliations:** 1 Center for Innovation in Medical Education, Leiden University Medical Center, Leiden, The Netherlands; 2 Department of Cardiology, Leiden University Medical Center, Leiden, The Netherlands; 3 Leiden University Graduate School of Teaching, ICLON, Leiden, The Netherlands; 4 Department of Medical Statistics, Leiden University Medical Center, Leiden, The Netherlands

**Keywords:** Active learning, Comprehension, Peer instruction, Self-explanation, Transfer, Physiological concepts

## Abstract

Comprehension of physiology is essential for development of clinical reasoning. However, medical students often struggle to understand physiological concepts. Interactive learning through Peer instruction (PI) is known to stimulate students’ comprehension, but its relative efficacy and working mechanisms remain to be elucidated. In this study, we investigated if and how PI could optimize comprehension of physiological concepts and transfer relative to Self-explanation (SE) which is considered a lower-order type of overt learning. First-year medical students (n = 317) were randomly assigned to either PI or SE in a pre-post test design, followed by a set of near and far transfer questions. In both PI and SE groups post-test scores were significantly improved (*p *< 0.0001) with PI outperforming SE (+ 35% vs. + 23%, *p *= 0.006). Interestingly, a substantial number of students with initial incorrect answers even had enhanced scores after discussion with an incorrect peer. Both methods showed higher transfer scores than control (*p* = 0.006), with a tendency for higher near transfer scores for PI. These findings support PI as a valuable method to enhance comprehension of physiological concepts. Moreover, by comparing the effects of interactive PI with constructive SE we have established new insights that complement educational theories on overt learning activities.

**Electronic supplementary material:**

The online version of this article (10.1007/s10459-018-9858-6) contains supplementary material, which is available to authorized users.

## Introduction

Comprehension of physiological concepts is considered one of the main gateways towards efficient medical training (Finnerty et al. 2010). Conceptual insight in physiology is profoundly valuable for recall and application of clinical knowledge and diagnostic accuracy (Goldszmidt et al. 2012; Kulasegaram et al. 2013; Lisk et al. 2016, 2017; Nielsen et al. 2012; Woods 2007; Woods et al. 2007). However, acquiring comprehension of physiological concepts underlying clinical cases appears problematic for many medical students (Michael 2007).

The presence of misconceptions is among the recurring arguments to explain the struggle that students experience in gaining conceptual understanding (Badenhorst et al. 2016; Feltovich et al. 1993; Michael 1998). These misconceptions may originate from various sources, including teaching methodology (Badenhorst et al. 2015). An interactive instructional design named Peer instruction (PI) has been able to improve understanding of scientific concepts (Brooks and Koretsky 2011; Lasry et al. 2016; Miller et al. 2014; Smith et al. 2009, 2011; Vickrey et al. 2015; Zhang and Henderson 2016). As developed by Mazur et al., PI contrasts with traditional lectures by including questions and subsequent peer discussions to uncover misconceptions and stimulate conceptual understanding among students (Crouch and Mazur 2001). In multiple studies PI has shown to enhance student performance on medical physiology exercises (Cortright et al. 2005; Giuliodori et al. 2006; Rao and DiCarlo 2000; Relling and Giuliodori 2015), but the exact mechanisms underlying the beneficial influence of PI on comprehension remain to be clarified. In particular, studies addressing these mechanisms through direct comparison of PI with other types of overt learning strategies are scarce. This has created a gap in our knowledge about the relative efficacy of PI and in our understanding of factors that lead to the superiority of interactive learning over other overt learning methods.

Overt learning activities typically encompass student-centered instructional designs through which engagement of students in the learning process is fostered (Bonwell and Eison 1991). The usefulness of such strategies to improve students’ comprehension and tackle misconceptions has been acknowledged by the educational research community (Anderson et al. 2011; Prince 2004). In order to clarify the relative effects of overt learning methods, Chi has developed the *differentiated overt learning activities* framework (Chi 2009). According to this framework, active learning is just one form of overt learning as one can distinguish *active learning* from *constructive learning* and *interactive learning*. *Active* strategies comprise of one’s engagement with the learning material without creating new knowledge (e.g. underlining sentences while reading). *Constructively* designed methods should promote generation of novel ideas that go beyond the presented information (e.g. Self-explanation), whereas *interactive* activities involve development of each other’s thoughts and consequent co-construction of knowledge (e.g. Peer instruction). Chi has confirmed the hypothesis that *interactive* activities are most effective for high level cognitive processing, followed by *constructive* and *active* learning respectively (Chi and Wylie 2014). Chi’s hypothesis has been supported by educational research in the fields of physics and engineering (Lasry et al. 2016; Linton et al. 2014; Menekse et al. 2013).

Chi emphasizes that the mechanisms through which interactive methods cause enhanced cognitive processing during concept learning are still unclear. Therefore, we can only hypothesize about these potential underlying processes. During constructive learning, such as Self-explanation (SE), students are instructed to individually generate self-explanations on conceptual problems. This type of learning is suggested to stimulate one’s knowledge organization and integration, which in turn is assumed to increase comprehension of the learning material since misconceptions may become more evident (Chi and Bassok 1989). The SE effect has been demonstrated in various experimental designs, including assessments that range from memory tasks to clinical reasoning performance (Chamberland and Mamede 2015; Dunlosky et al. 2013). Perhaps interactive learning is anticipated to be more effective than constructive learning because learners are provided with an opportunity to compare and contrast their knowledge with others, thereby establishing a more robust representation of the learned concept. Additionally, learners can build on each other’s line of reasoning, a process which Chi refers to as *co*-*construction*. Note that interactive learning is thus also constructive in nature, but additionally allows individuals to expand upon their peers’ thoughts and ideas. Knowing if and how an interactive strategy such as PI gains higher performance levels compared with individual constructive learning methods could help us optimize teaching and learning in medical physiology education.

The main objective (research question #1) of this study is to investigate the efficacy of interactive PI learning on comprehension of physiological concepts compared with individual constructive SE. Based on Chi’s conceptual framework and a previous study from Lasry and colleagues who show superiority of PI over individual reflection in physics education, we expect that PI will outperform SE (Lasry et al. 2016). A second objective (research question #2) is to explore the specific influence of dyad composition in the PI groups to get a better grip on the working mechanisms of interactive learning. We hypothesize that correct individuals will generally have a positive influence on their peer. Additionally, dyads that consist of students who both have incorrect answers may still increase their comphrension due to the opportunity of comparing and contrasting their thoughts and ideas, leading to potential co-construction of knowledge. Such co-construction may also lead to enhanced transfer of knowledge. Transfer has shown to be a substantial challenge for students (Kulasegaram et al. 2012, 2015), but the ability to apply knowledge to novel contexts is essential for developing useful clinical reasoning skills (Woods 2007). However, the effect of PI and SE on transfer performance in medical physiology has not been addressed in previous research. Our third objective (research question #3) is therefore to study the effect of PI and SE on transfer performance as this provides insight in the extent to which constructive and interactive methods can promote learning. Based on the idea of co-construction, we hypothesize that if these learning strategies would promote comprehension, then transfer of knowledge may enhance accordingly. In all, findings on the relative efficacy of PI, its working mechanisms, and its influence on transfer will add valuable information to the active-constructive-interactive framework. Moreover, outcomes may enable researchers and educators to optimize instructional designs for concept learning in medical education.

## Methods

### Participants and setting

This study was conducted in a cohort of first-year medical students (N = 321) at the Leiden University Medical Center (LUMC). The study protocol was implemented in a compulsory, 8-weeks course on integrative cardiovascular, respiratory and kidney physiology. The protocol was executed in two different mandatory supervised seminars (24 groups, 12–14 students/group). Allocation to the groups was arbitrarily except for the aim to have a similar female/male ratio in all groups.

Although the seminars were mandatory, enrolment in the study was on a voluntary basis as students autonomously decided if their answers could be used for research purposes by signing the informed consent form. Students were informed that data analyses would be performed anonymously and that they could withdraw their consent at any given time. Moreover, they were ensured that the results would not affect their course grades. Students did not receive any additional credit for their participation. The study protocol was reviewed and approved by the LUMC institutional scientific committee on educational research.

### Study design

**Fig. 1:**
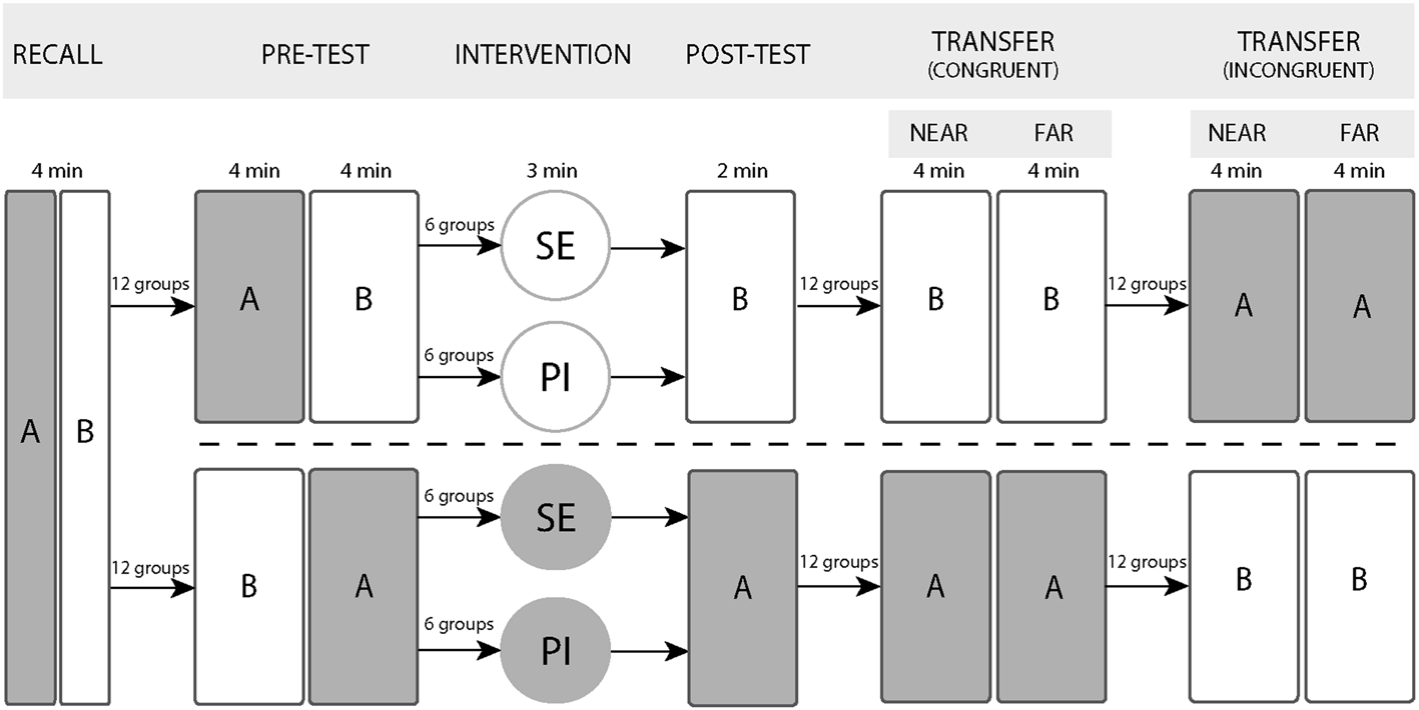
Study design

The study protocol was conducted in two different seminars spaced two weeks apart, the study flow chart is shown in Fig. 1. Each seminar group was allocated to a specific intervention. In the PI groups, students were coupled with a peer to form dyads. Students were instructed to discuss their answers on the specified concept with their peer. The formation of dyads was randomized using a pseudo-random number generator and investigators were blinded to the allocation. In the SE condition, students were instructed to reflect critically on their answers in silence using the summary sheet to generate explanations and optionally write down their thoughts.

At the start of the seminar, students received a sheet (Supplementary S1) summarizing two physiological concepts, i.e. resistance (concept A) and compliance (concept B), that were discussed in previous plenary lectures. After reviewing the information, students individually answered a set of four multiple-choice (MC) recall questions, i.e. recall test, consisting of two concept A and two concept B questions. Performance on these questions was used to compute an individual baseline score. Then, students had to individually answer four MC questions, two related to concept A and two to concept B (i.e. *pre*-*test*). After the pre-test, they were assigned to an intervention (PI or SE) which focussed on only one of the two concepts followed by the same MC questions that students had to re-answer individually (i.e. *post*-*test*). By applying the intervention to only one of two concepts, an internal control for each participant was established. Subsequently, two near transfer and two far transfer questions on both concepts were performed by all students. In this study design, transfer questions are classified as *congruent* or *incongruent* to the intervention depending on one’s learning intervention trajectory during the experiment. For example, a near transfer question on concept A was classified as *congruent* for all individuals that received PI or SE on concept A and *incongruent* for those receiving PI or SE on concept B. Finally, the seminar continued with a class-wide, teacher-supervised discussion and explanation of all 16 questions.

## Materials

Questions were framed in accordance with the revised version of Bloom’s taxonomy of the cognitive domain; recall and comprehension questions for baseline testing, and application questions for comprehension and transfer testing (Anderson et al. 2001). Transfer questions involved similar contexts, i.e. near transfer, and novel contexts that had not been addressed during the lecture or seminar, i.e. far transfer (Barnett and Ceci 2002). The information sheet and questions used in the protocol were designed by an expert physiologist (PS). All questions used in this study were derived from a database containing previous exam questions.

### Outcome measures and statistics

IBM SPSS Statistics Version 23.0 (IBM Corp., Armonk, New York, USA) and GraphPad Prism Version 7.02 (GraphPad Software, La Jolla, California, USA) were used for all data analyses and visualizations. Descriptive statistics are provided as means and standard errors of the mean, unless otherwise mentioned.

#### Baseline knowledge

A recall test was used to compute baseline knowledge. Comparison of the average recall scores, with a maximum score of 4 points, in the total of 24 groups would reveal possible differences in baseline knowledge prior to intervention. Similar baseline knowledge between groups ensures that intervention effects on comprehension and transfer are not due to differences in baseline performance prior to intervention. A one-way ANOVA was conducted including SE_A_, SE_B_, PI_A_, and PI_B_ as experimental groups to test baseline differences.

#### Comprehension

A comparison of post-test  scores between PI and SE groups provides an indication of the relative effect of each intervention on students’ comprehension. We applied an ANCOVA to determine the effect of PI and SE intervention on the post-scores, correcting for students’ pre-test scores.

#### Peer influence

The specific influences of peers on comprehension in the PI group were assessed by two analyses. Firstly, we examined the probability of staying or turning correct after discussion with either an incorrect or correct peer. Outcomes were compared to SE to measure the relative effect. Secondly, we examined the change in different dyad compositions, i.e. both correct (C–C), one correct (I–C), both same answer incorrect (I–I) or both different answer incorrect (I–I*). Dyad compositions before and after PI intervention were analysed to determine the effect of peer–peer interaction on post-test performance. Computing the prevalence of these dyad compositions before and after PI intervention indicated the influence of an incorrect or correct peer on post-test performance of a participant. A Chi square test of independence was performed to determine differences in dyad composition before and after PI intervention. Continuity correction was applied for 2 × 2 contingency tables.

#### Transfer

Transfer was assessed by measuring students’ performance on near and far transfer questions. All students received questions on two concepts, i.e. concept A and B, but the intervention (PI or SE) was applied to only one of these concepts. The comparative effect of PI and SE on transfer was assessed by measuring the difference in performance scores between both interventions on *congruent* questions. Additionally, the absolute effect of each intervention was computed by comparing students’ performance on *congruent* and *incongruent* questions. A generalized estimating equations (GEE) approach was used to analyze performance on transfer exercises corrected for multiple comparisons and repeated measures. In this model, scores on near and far transfer questions were predicted based on the intervention (SE-PI), type of transfer (near-far) and protocol trajectory (congruent-incongruent).

## Results

A total of 321 first-year medical students from Leiden University Medical Center were enrolled in the medical physiology course, of whom 317 students consented to participate in the study (98.7%).

### Comprehension

Results on the recall test prior to intervention showed similar performance outcomes between the four experimental groups [SE_A_ (n = 83), SE_B_ (n = 82), PI_A_ (n = 78), PI_B_ (n = 74)] with an overall average score of 2.37 ± 0.06 out of 4 points. Analysis of variance indicated no significant between-group differences (F_(3313)_ = 0.751, *p* = 0.522).

**Fig. 2:**
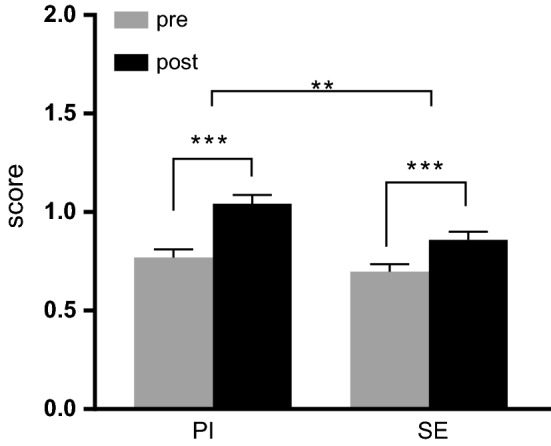
Average score on pre and post tests by condition. ***p < 0.0001, **p = 0.006

Total scores (max. 2 points) for the pre-test and post-test were computed for each student, data of the two seminars were combined to compute average scores (Fig. 2). The pre-test scores were similar in the PI and SE groups (PI: 0.77 ± 0.04 vs. SE: 0.70 ± 0.04, t_(620)_ = 1.295, *p* = 0.196) and increased on the post-test (PI: 1.04 ± 0.05 vs. SE: 0.86 ± 0.04, both *p *< 0.0001). In the PI group the average score increased by 0.27 (CI: 0.20–0.35) and in the SE group by 0.16 (CI: 0.09–0.23), indicating a greater improvement of scores in the PI group (F_(1619)_ = 7.671, *p* = 0.006; η_p_^2^ = 0.012). The performance gain in PI groups was shown for all questions (see Supplementary, S2). Additionally, a control analysis showed that the outperformance of PI vs SE resulted from both more incorrect-to-correct changes and fewer correct-to-incorrect switches by individual students (see Supplementary, S3).

### Peer influence

**Fig. 3:**
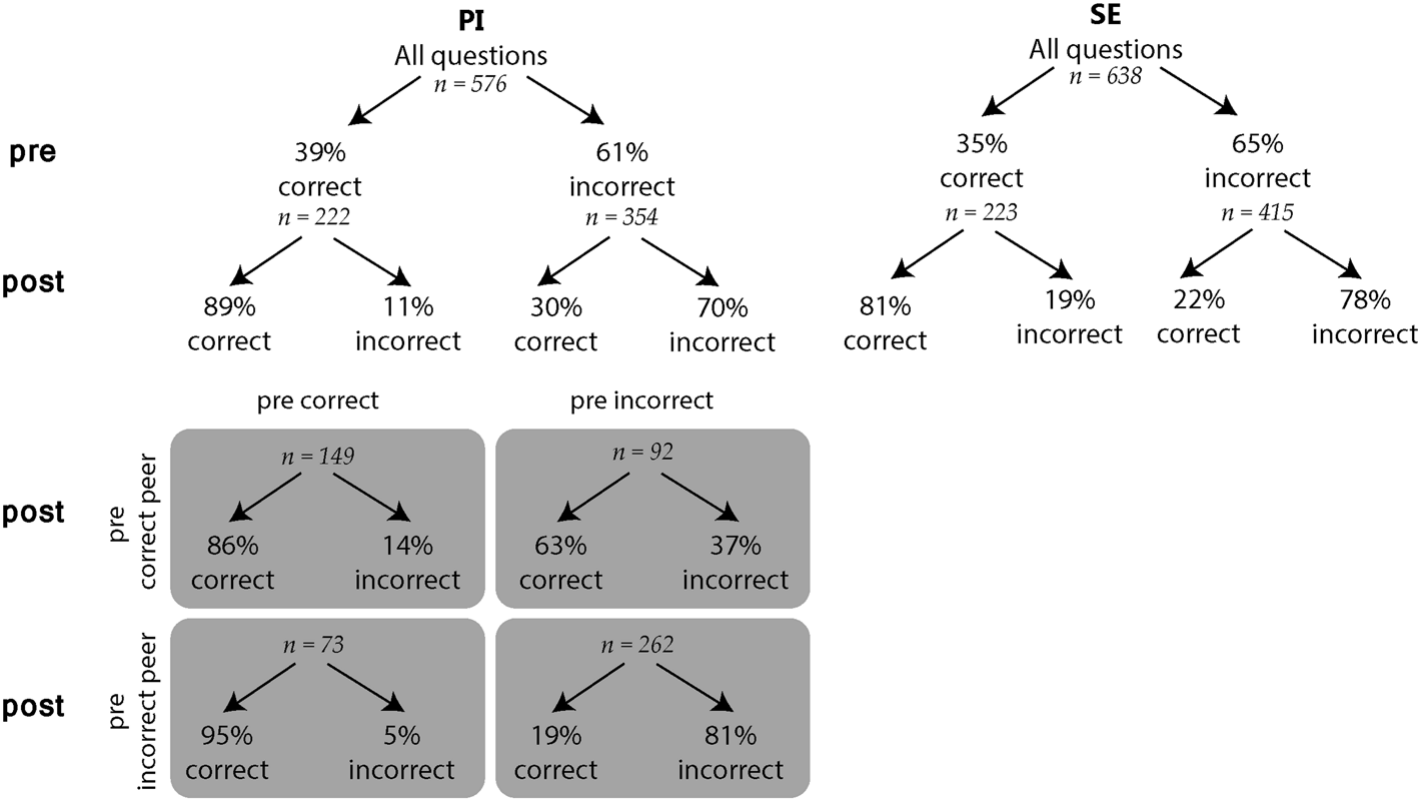
Students’ responses on the pre- and post-test. The quadrant below visualizes the answer changes for PI groups, categorized for peers that were either correct or incorrect prior to intervention

The efficacy of PI presumably depends on whether you are coupled with a peer who was initially either correct or incorrect (Fig. 3). A students’ initial correct answer stayed correct more often when coupled with an incorrect peer (95%) versus coupled with an correct peer (86%). In both cases, the chance of staying correct was higher than with SE (81%). A student with an initial incorrect answer turned to a correct answer more often when coupled with a correct peer (63%) versus when coupled with an incorrect peer (19%). With SE, 22% of initially incorrect answers were followed by a correct answer on the post-test.

**Fig. 4:**
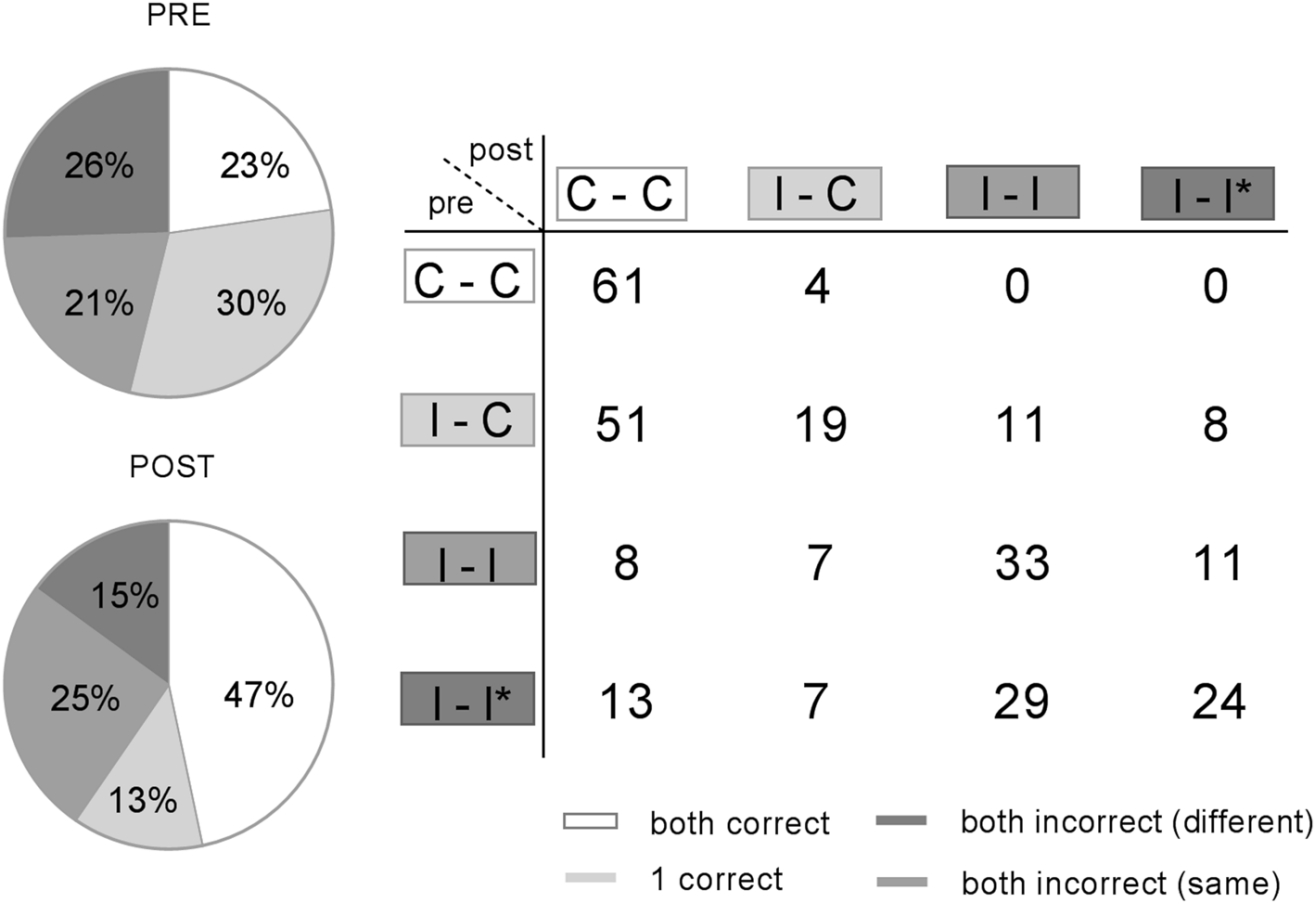
Dyad compositions in the PI group on the pre- and  post-test

Additionally, we investigated the dyad compositions to examine the influence of peer–peer interaction on post-test performance in more detail (Fig. 4). There was a difference in dyad answer composition pre-intervention compared to post-intervention (*Χ*^2^_(9)_  = 145.799, *p *< 0.001; φ = 0.714). Post-intervention there were more C–C dyads (pre: 23% to post: 47%, t_(285)_ = 8.776, *p *< 0.0001). This was due to 57% of initial I-C dyads, 14% of initial I–I dyads and 18% of initial I–I* dyads that became C–C dyads after peer discussion. Also, 12% of I–I dyads and 10% of I–I* dyads became half correct, i.e. I–C, on the post-test. Furthermore, there were 6% of initial C–C dyads that changed to I-C dyads, and 12% and 8% of I-C dyads that changed to I–I or I–I* respectively.

### Transfer

Students scored significantly higher on near transfer questions as opposed to far transfer questions (Table 1; *p *< 0.0001) On both near transfer (PI: 0.95 ± 0.04; SE: 0.89 ± 0.04) and far transfer (PI: 0.80 ± 0.04; SE: 0.82 ± 0.04) tests, no significant differences in scores were found between the groups (B = 0.004, se = 0.035, *p* = 0.904). Overall, students performed significantly better on congruent questions versus incongruent questions (*p *= 0.006). For near transfer, the difference between scores on congruent and incongruent questions (i.e. intervention versus no intervention) was 0.112 ± 0.05 for PI and 0.034 ± 0.05 for SE. For far transfer, the difference was 0.040 ± 0.05 for PI and 0.085 ± 0.04 for SE.

**Table 1:** Predictors of transfer performances

Parameter	*B*	SD	95% CI
Threshold	0.353	0.035	[0.000, 0.284]
Type of transfer	− 0.101^***^	0.026	[− 0.151, − 0.051]
Intervention	0.004	0.035	[− 0.065, 0.073]
Protocol trajectory	0.069^**^	0.025	[0.020, 0.117]
Type of transfer × intervention	− 0.010	0.026	[− 0.060, 0.041]
Intervention × protocol trajectory	0.006	0.025	[− 0.043, 0.055]
Type of transfer × protocol trajectory	− 0.008	0.024	[− 0.056, 0.040]

Dependent variable: answer, incorrect (0) or correct (1). Covariates: Type of transfer, near (− 1) or far (1); Intervention, SE (− 1) or PI (1); Protocol trajectory, incongruent (− 1) or congruent (1)

**p < 0.01, ***p < 0.0001

## Discussion

With this study we aimed to investigate the relative influence of interactive learning compared with individual constructive learning on the comprehension and transfer of physiological concepts in medical education. We found that interactive Peer Instruction (PI) and constructive Self-explanation (SE) both enhanced students’ comprehension. The beneficial effects were more pronounced in the PI group, indicated by higher scores on the post-test and higher scores on near transfer questions. On transfer tests, interactive learning also showed enhanced performance versus the control condition, but in this respect no significant difference was found between PI and SE.

### Comprehension

This study has shown that the implementation of interactive PI in a medical physiology course significantly improved students’ performance on conceptual physiology exercises. A head-to-head comparison of PI with SE demonstrated increased comprehension of students after PI (Fig. 2). In addition, analysis of answer changes before and after intervention showed that PI resulted in more incorrect-to-correct switches and less correct-to-incorrect switches, yielding an overall beneficial outcome (Fig. 4, Figure S3). Results are in accordance with a previous study of Miller et al., who showed that the majority of answer changes due to PI is from incorrect-to-correct (Miller et al. 2015).

The use of active learning strategies has repeatedly illustrated to facilitate student performance and conceptual understanding in science education (Chamberland and Mamede 2015; Freeman et al. 2014; Prince 2004). According to the theoretical framework established by Chi et al., active learning can be subdivided into three categories (i.e. interactive, constructive and active learning) (Chi 2009; Chi and Wylie 2014). Chi’s active learning hypothesis has already received support from experimental studies in science education (Menekse et al. 2013), and is reinforced by our findings suggesting that co-construction of knowledge through PI enhances comprehension more than individual constructive SE.

### Peer influence

As the effect of PI on post-test performance presumably depends on the type of peer (i.e. correct or incorrect), answer changes on the pre-post-test were investigated from the peer-perspective. According to our results, a student who was initially correct benefitted more from discussion with an incorrect peer compared with a correct peer (Fig. 3) and in both cases the results were better, i.e. fewer correct- to-incorrect switches, than after SE. In case a student was initially incorrect, PI with a correct peer lead to significantly more incorrect-to-correct switches than SE, and PI with an incorrect peer showed similar effects as SE. These outcomes imply that an interactive learning strategy such as PI can be preferred over constructive strategies, since even discussion with an incorrect peer yields similar or increased performance in direct comparison with SE. In agreement with Chi’s hypothesis of co-construction, we suggest that an interaction between a correct and an incorrect student might yield more contrasting thoughts or ideas which drives a fruitful discussion and stimulates deeper understanding. This line of reasoning can be complemented by conceptual change research, advocating that awareness of potential misconceptions leads to increased comprehension (Piaget 1978; Posner et al. 1982).

The influence of peer–peer interaction on comprehension was also investigated on the dyad level (Fig. 4). A vast majority of dyads who were initially correct stayed correct after peer discussion and half correct dyads were able to convince their incorrect peer in most cases. Moreover, initially correct dyads never became an incorrect dyad after intervention, illustrating that students were positively influenced by their correct peer, rather than negatively influenced by their incorrect peer. Mazur supports this statement as he concluded in earlier studies that it is easier to change the mind of an individual who has an incorrect answer than it is to change the mind of an individual who initially chose the correct answer for the right reasons (Mazur 1997).

Interestingly, a substantial number of dyads in which both peers initially had an incorrect response were still able to both provide the correct answer to the question after PI (Fig. 4). We demonstrated a positive effect of peer–peer interaction on performance outcomes that was also present in dyads of which both students initially had an incorrect answer. When students had a different incorrect answer compared with their peer, their chances of providing the correct answer was slightly higher compared to students and their peers who had the same incorrect answer. These results complement previous research by Smith et al., who illustrated improved conceptual understanding after PI in a genetics course independent of students’ answers in the discussion group (Smith et al. 2009). In their study, a large percentage of students reported that even incorrect students could learn from each other’s reasoning as an individual could figure out the answer by talking through the question with another peer that does not know the answer either. Furthermore, a discussion with two incorrect individuals would stimulate comprehension as all options are explored and the ones that cannot be correct are eliminated. One could assume that such discussions are more explorative when students have different incorrect answers which might explain the more pronounced positive effect of peer discussion in disagreeing incorrect dyads compared with dyads consisting of two students with the same incorrect answer.

### Transfer

We included near and far transfer questions in this study to explore the effects of overt learning interventions on transfer of conceptual knowledge. Students’ performance on near and far transfer assignments did not differ significantly between PI and SE intervention (Table 1). However, in all conditions there was a better performance on transfer questions congruent to an interactive or constructive learning trajectory compared to conceptual questions on which students did not receive any intervention. This finding is in line with earlier studies reporting a positive influence of overt learning on transferring conceptual knowledge and with other studies investigating transfer in science education (Cortright et al. 2005; Kulasegaram et al. 2017; Smith et al. 2009).

Although the effects did not reach significance, the SE groups tended to perform best on the far transfer test. SE has shown to have a beneficial influence on transfer performance in previous studies (Dunlosky et al. 2013). Interestingly, the effect increases when the tasks become more complex (Chi et al. 1994; Wong et al. 2002). Possible explanations for this observation can be derived from schema formation theory, stating that SE may enhance the connectedness of inter- and intra-schema components, thereby stimulating comprehension (Mayer 1975).

One could argue that the higher scores on the post-test after PI versus SE result from the mere fact that two individuals might simply know more than one. However, our findings on the near transfer test suggest that, on average, students have gained better comprehension with PI since there is a tendency towards increased performance after peer discussion. Moreover, if only the ‘two-heads-are-better-than-one’ effect would cause the score increases on the post-questions, one would expect that the positive influence of PI would be less pronounced for difficult questions since fewer students can be matched with a correct peer. However, this argument is not supported by our analyses as PI shows a beneficial effect for all questions (Supplementary S2). Thus, other mechanisms seem to contribute to the potential of PI to enhance comprehension. In addition to the co-construction hypothesis, incorrect students may gain insight in their reasoning flaw while explaining their answer to their peer, and for correct students PI may act upon the ‘teaching is learning’ rationale.

### Limitations

This study has concentrated on developing a controlled design for testing the effects of learning strategies on comprehension and transfer in a classroom setting. Due to practical considerations regarding feasibility, a limited number of questions per seminar (16 in total) were included in the protocol. In order to generalize the efficacy of active learning in medical physiology education, more concepts should be examined in a variety of near and far transfer settings. Another consequence of the chosen study design is the careful interpretation of the practical relevance of significant outcomes. An increase in test scores of 0.27 (13.5%) after PI intervention may seem minor, but is considered robust due to the large sample size and the various control conditions that were implemented in the protocol. Also, the benefits of peer discussion may become more compelling in an open question setting and by increasing the number of included questions and concepts. Lastly, although the supervising teachers were briefed and instructed to maintain exam conditions and followed a written protocol, a full guarantee that all students worked individually on the questions when they were instructed to do so cannot be given. Moreover, we cannot guarantee that each student in the PI group used the scheduled time between pre-post testing to actively discuss with their peers nor do we know if all students in the SE groups were actually generating explanations for their answers.

### Summary and future directions

To our knowledge this study is the first to demonstrate the efficacy of PI on student performance on physiological concept questions compared with an individual constructive learning activity in medical students. Other studies have shown the additional value of PI in the classroom, but often did not address its relative effect (Cortright et al. 2005; Giuliodori et al. 2006; Rao and DiCarlo 2000; Smith et al. 2009; Vickrey et al. 2015; Zhang and Henderson 2016). Moreover, PI protocols are usually restricted to analysis on a group level, not confirming if the reported effects are indeed due to an increase of performance in the majority of participants (Cortright et al. 2005; Lasry et al. 2016; Rao and DiCarlo 2000; Zhang and Henderson 2016). By performing analyses on various levels we showed the more specific influences of PI on performance outcomes. The conclusion that even incorrect groups can achieve a correct answer after PI is in accordance with current literature (Relling and Giuliodori 2015; Smith et al. 2009). Furthermore, research on this topic generally does not report on students prior knowledge on the concepts of interest. By including a baseline knowledge test, we controlled for the influence of students prior knowledge on performance outcomes. Lastly, by assessment of students’ performance on near and far transfer questions we investigated if the effect of overt learning interventions was transferable to novel assignments.

Future research on the efficacy of PI in medical education may include the use of open questions to gain insight in the influence of discussion on causal reasoning processes. Studies may also consider the use of between and within subject control conditions as presented in this research. Extended study protocols, including more questions, concepts and follow-up tests will provide a more accurate indication of the long-term efficacy of PI on comprehension. Also, a qualitative approach would provide additional insight in the personal thinking and reasoning processes of students and the valuable aspects of peer discussion (Brooks and Koretsky 2011; James and Willoughby 2011; Nielsen et al. 2016; Wood et al. 2014). From a theoretical point of view we propose to consider the use of other theories, such as schema formation, to further uncover the working mechanisms of interactive learning strategies. Lastly, we believe that the absolute performance of students on medical physiology exercises needs further investigation. In our experiment, observations of the absolute scores indicate that a large percentage of students is incapable of achieving full conceptual understanding. Therefore, uncovering best practices or refinement of existing methods for learning and understanding medical physiological concepts should be studied in future work.

## Conclusion

This study demonstrates the effect of active learning strategies on understanding and transfer of physiological concepts in the medical curriculum. In particular, interactive learning activities such as PI show their value for designing effective teaching methods in medical physiology education. Future research may elaborate on the working mechanisms of interactive learning activities in gaining conceptual understanding. Moreover, additional research may uncover the value of these learning strategies for transfer of conceptual knowledge to the clinical practice.

## Electronic supplementary material

Below is the link to the electronic supplementary material.

Supplementary material 1 (DOCX 533 kb)
