# Supplementary material for: Peer instruction improves comprehension and transfer of physiological concepts: a randomized comparison with self-explanation
Source: Adv Health Sci Educ Theory Pract. 2018 Oct 20;24(1):151–65. doi: 10.1007/s10459-018-9858-6 (PMC6373526; doi:10.1007/s10459-018-9858-6)
Supplement: Supplementary file 1 — Supplementary material 1 (DOCX 533 kb) [file 10459_2018_9858_MOESM1_ESM.docx]

**SUPPLEMENTARY**

Figure S1 is the information sheet summarizing the concepts handled in previous lectures and self-study that was provided to the students at the beginning of the study protocol, prior to the recall test. Students were given two minutes to study the information sheet before continuing with the recall test. All information is provided in Dutch.


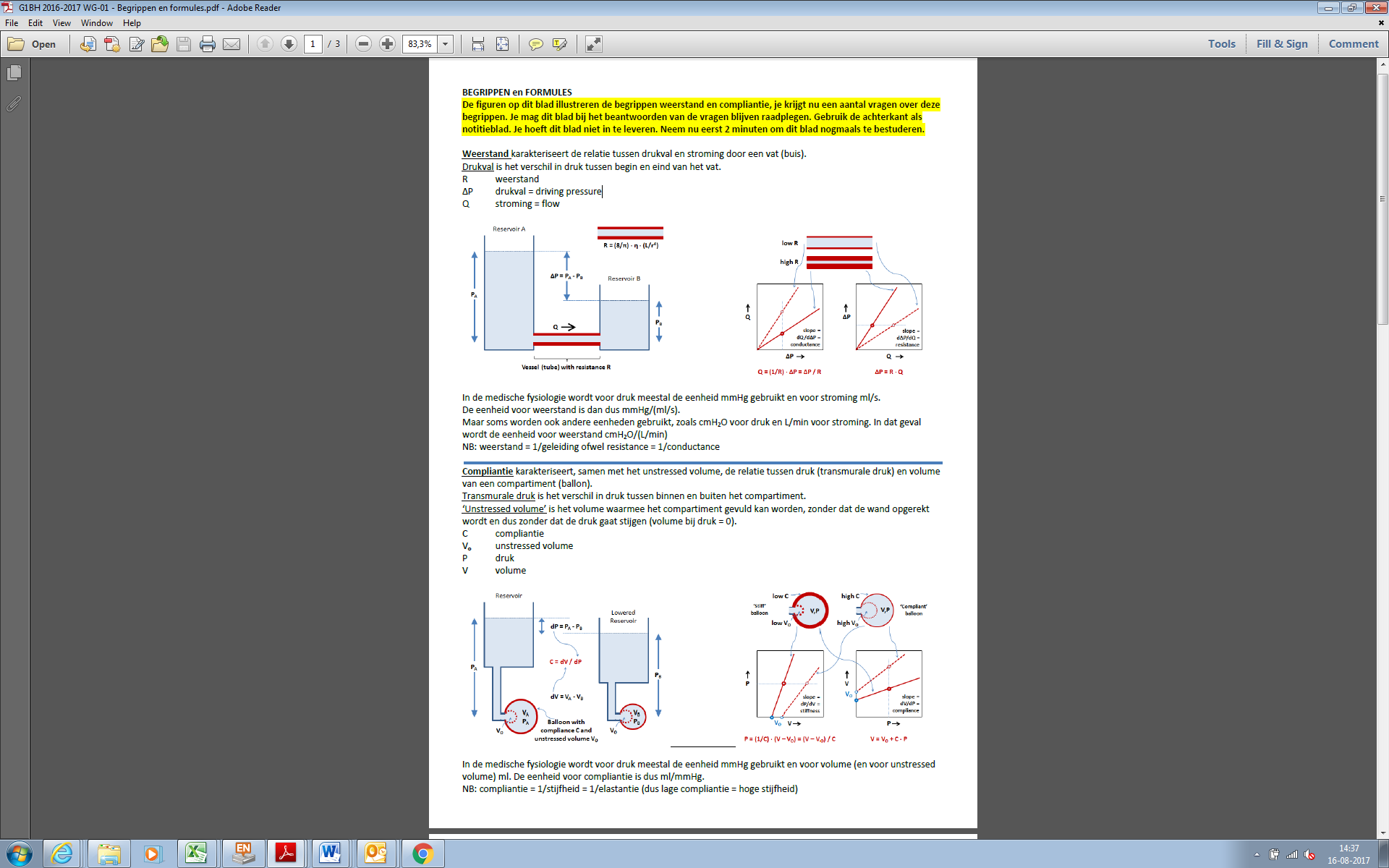


**Figure S1 | Information sheet included in the study protocol (Dutch).**

**Figure S2 | Pre-test scores on conceptual problems in seminars 1 and 2.** Pre-post scores were examined separately for all four questions (two concepts in two seminars) to assess if efficacy of active learning was consistent among these exercises. Average pre scores are shown for PI and SE groups (left panel). Lowest scores were found for exercise B_s2_. The relative change in scores was positive for all exercises in both SE and PI groups, with higher gains for PI in all cases (right panel). Accordingly, a significant increase of 0.17 was found on exercise B_s2_ scores in the second seminar between PI and SE groups (t_(151)_=3.206, *p* = 0.023). The highest performance gains for single exercises were observed in the PI condition, showing the largest increase of 0.52±0.09 for A_s2_.


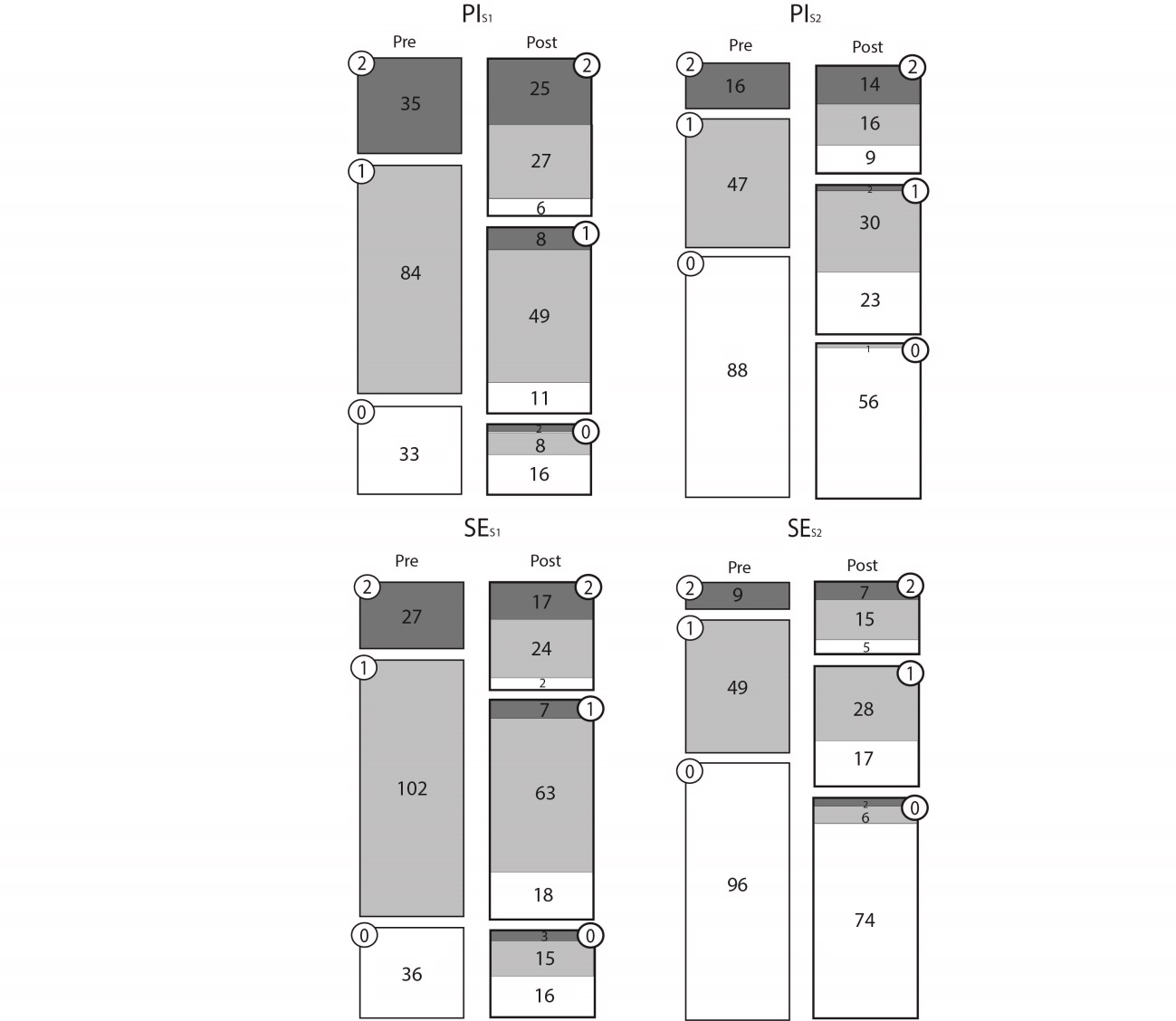


**Figure S3 |** **Students’ individual scores on conceptual problems before and after intervention.** The conceptual problems consisted of two related questions each yielding 1 point, thus a maximal score of 2. This scheme shows the distribution of total scores pre and post intervention for all students participating in the protocol. The total scores (0, 1 or 2 points) are indicated in the circles. The white, grey and dark areas indicate, respectively, students with a pre-test score of 0, 1 or 2 points. The numbers in the bars indicate the number of students with a specific score. Data for both seminars is depicted separately to illustrate the difference in difficulty level between the seminars. Seminar 2 had more students with score 0 and fewer with scores 1 or 2, but similar shifts towards higher scores post intervention.
